# Effect of the coughing technique during subcutaneous heparin injection on pain severity and individual satisfaction

**DOI:** 10.1590/1518-8345.6504.3924

**Published:** 2023-05-15

**Authors:** Dilek Yılmaz, Dilan Ayhan, Derya Uzelli Yılmaz, Fatma Düzgün

**Affiliations:** 1 Bursa Uludağ University, Faculty of Health Sciences, Department of Nursing, Bursa, Turquía.; 2 Bursa Uludağ University, Health Application and Research Center, Bursa, Turquía.; 3 İzmir Katip Çelebi University, Faculty of Health Sciences, Department of Nursing, İzmir, Turquía.

**Keywords:** Coughing Technique, Non-Pharmacological Method, Nursing, Pain, Patient Satisfaction, Subcutaneous Heparin Injection, Técnica de Toser, Método no Farmacológico, Enfermería, Dolor, Satisfacción del Paciente, Inyección Subcutánea de Heparina, Técnica de Tosse, Método não Farmacológico, Enfermagem, Dor, Satisfação do Paciente, Injeção Subcutânea de Heparina

## Abstract

**Objective:**

to examine the effect of the medium intensity coughing technique during subcutaneous low molecular weight heparin injection on pain severity and individual satisfaction in general surgery patients.

**Method:**

the prospective, quasi-experimental study included 100 patients who had been prescribed a subcutaneous low molecular weight heparin injection once in 24 hours. Each patient received two injections by the same researcher, one using the standard injection technique with medium intensity coughing technique and the other only the standard injection technique.

**Results:**

there was a statistically significant difference between patients’ mean scores on pain severity and satisfaction levels after injections administered by the two techniques (p= 0.000). Also, it was found that gender affected pain severity relating to the injection but did not affect the level of individual satisfaction.

**Conclusion:**

the medium intensity coughing technique was found to reduce pain severity and increase patient satisfaction in general surgery patients receiving subcutaneous low molecular weight heparin injections. Trial registration: NCT05681338.

## Abstract (es)

**Objetivo:**

examinar el efecto de la técnica de tos de mediana intensidad durante la inyección subcutánea de heparina de bajo peso molecular sobre la severidad del dolor y la satisfacción individual en pacientes de cirugía general.

**Método:**

estudio prospectivo, cuasi experimental, que incluyó a 100 pacientes a los que se les prescribió una inyección subcutánea de heparina de bajo peso molecular una vez cada 24 horas. Cada paciente recibió dos inyecciones por el mismo investigador utilizando técnica de inyección estándar con técnica de tos de intensidad media y solo técnica de inyección estándar.

**Resultados:**

hubo una diferencia estadísticamente significativa entre las puntuaciones medias de los pacientes sobre la intensidad del dolor y los niveles de satisfacción después de las inyecciones administradas por las dos técnicas (p= 0,000). Además, se encontró que el género afectó la severidad del dolor relacionado con la inyección, pero no afectó el nivel de satisfacción individual.

**Conclusión:**

se encontró que la técnica de tos de intensidad media reduce la severidad del dolor y aumenta la satisfacción del paciente de cirugía general que reciben inyecciones subcutáneas de heparina de bajo peso molecular. Registro del ensayo: NCT05681338.

## Abstract (pt)

**Objetivo:**

examinar o efeito da técnica de tosse de média intensidade durante injeção subcutânea de heparina de baixo peso molecular na intensidade da dor e satisfação individual em pacientes submetidos à cirurgia geral.

**Método:**

estudo prospetivo, *quasi experimental* que incluiu 100 pacientes que haviam recebido uma injeção subcutânea de heparina de baixo peso molecular em 24 horas. Cada paciente recebeu duas injeções pelo mesmo pesquisador usando a técnica de injeção padrão com técnica de tosse de média intensidade e apenas técnica de injeção padrão.

**Resultados:**

houve diferença estatisticamente significativa entre as pontuações médias dos pacientes quanto à gravidade da dor e níveis de satisfação após as injeções administradas pelas duas técnicas (p = 0,000). Além disso, verificou-se que o sexo do paciente afetou a intensidade da dor relacionada à injeção, mas não afetou o nível de satisfação individual.

**Conclusão:**

a técnica de tosse de média intensidade reduz a intensidade da dor e aumenta a satisfação de pacientes submetidos à cirurgia geral recebendo injeções subcutâneas de heparina de baixo peso molecular. Registro do ensaio clínico: NCT05681338.

**Highlights:**

**(1)** Subcutaneous heparin injections cause pain at the injection site.

**(2)** Nurses are often undecided on the use of effective techniques in pain management.

**(3)** There is a need for an effective technique which is easy and simple to use.

**(4)** The use of the coughing technique is effective in subcutaneous heparin injections.

**(5)** The medium intensity coughing technique can easily be used to reduce the pain.

## Introduction

Today, medications are given by the oral and parenteral routes. One method of parenteral drug administration is subcutaneous injection, the method of introducing the medications under the dermis into the adipose tissue. The types of medication generally administered by the subcutaneous route are vaccinations, insulin, hormones and low molecular weight heparin (LMWH)^(1)^. LMWH is a type of heparin which is prepared by the depolymerization of the widespread form of heparin, and can only be given by the subcutaneous route^(2)-(3)^. LMWH is frequently used as a treatment and particularly as a protective in clinical situations where there is a risk of thromboembolism or where thromboembolitic events have occurred^(3)-(4)^. LMWH has advantages such as high bioavailability, a powerful antithrombotic effect and a lowered risk of bleeding, making it increasingly preferred in clinical applications^(5)^.

Generally occurring local side effects of the administration of LMWH by the subcutaneous route are reported to be bruising, hematoma and pain at the injection site. One complication frequently complained of by patients is pain; this is caused by the presence of pain receptors in the subcutaneous tissue^(6)-(11)^. Also, a sensation of pain occurs as a result of tissue damage occurring with the introduction into the tissue of the LMWH solution^(12)^. This can cause adverse effects in the patient such as anxiety, a disturbance in body image, pain in the injection area, refusal of treatment, and even a loss of trust in the nurses^(7)^. For this reason, it is of great importance to implement strategies that will be of use in protecting patients from these kinds of adverse effects during the administration of subcutaneous heparin^(12)^.

In health institutions, one of the most important responsibilities of nurses is the safe administration of medications^(13)^. It is reported that after subcutaneous heparin injections, various factors can cause complications at the injection site^(14)^. In particular, factors which can lead to pain complications are seen to mostly arise from the injection technique, and there are studies evaluating non-pharmacological methods for managing pain developing in connection with subcutaneous heparin injection. These studies recommend techniques such as applying manual pressure to the injection area^(9),(12)^, extending the duration of the injection or injecting slowly^(15)-(16)^, using the ShotBlocker (Bionix, Toledo, OH, USA) apparatus^(6),(17)^, and cold application to the injection site^(2),(18)^.

Recently, the use of the coughing technique as a different non-pharmacological method in the management of pain developing from various invasive procedures has attracted attention. It is underlined that this technique is easy to learn, does not take time, and does not involve extra cost or equipment^(19)-(21)^. It is reported that its potential mechanism relies on the Valsalva maneuver and on directing the attention elsewhere^(22)-(23)^.

The coughing technique increases intrathoracic pressure and stimulation of the autonomic nervous system, thereby causing an increase in heart rate and blood pressure, a higher level of pressure in the subarachnoid space and baroreceptor activation^(20)-(21)^. The increase in pressure in the subarachnoid space brings the segmental pain prevention pathways into action, and this has the effect of reducing the perception of pain^(20),(22)^. It has also been reported that as a result of the Valsalva maneuver, this technique has an antinociceptive effect, reducing the perception of pain in connection with vagal nerve stimulation. During this maneuver, the vagus nerve is stimulated in connection with baroreceptor activation, and this has an antinociceptive effect^(24)-(27)^. Also, the antinociceptive effect has been explained as occurring through central inhibition relating to the noradrenergic, serotogenic and endogen opioid systems with vagus nerve stimulation^(23),(28)^. On the other hand, it has been reported that another mechanism of the coughing technique depends on the method of directing the attention elsewhere and of diverting the attention so that the person does not feel pain^(29)^, and that it increases a person’s tolerance of pain^(23),(30)^. Studies in the literature dealing with the coughing technique report that it is effective in reducing pain in interventions such as peripheral intravenous catheterization^(22)-(23)^, taking a blood sample^(20)^, or vaccination^(19),(21)^.

In observing the administration of subcutaneous LMWH injection administration in the clinical setting, it is seen that there is much indecision on the part of nurses as to which technique should be used to increase satisfaction and to manage the pain which develops in relation to this frequently administered injection. In particular, in addition to factors such as an excessive amount of work, a large number of patients and a shortage of time, it was observed that there was a big gap in knowledge of the use of an effective technique which was easy for nurses to use, was simple, and did not involve equipment or cost, to increase individual satisfaction and reduce the pain relating to subcutaneous LMWH injections. Recently, despite reports that the coughing technique is effective in controlling the pain arising from invasive procedures^(19),(22)-(23)^, it was seen that the effect of this technique had not been investigated in the administration of subcutaneous LMWH injections. Also, it was seen that there were no studies considering this technique along with individual satisfaction, which is a very important parameter in increasing patient trust. Thus, a need was felt for this study, as there were few on this topic. It is thought that the results of the study will help nurses to give injections safely while reducing the pain of subcutaneous LMWH injections and increasing satisfaction with them. This study may serve as a guide for future researchers, who can use this research as a reference. Accordingly, the aim of this study was to examine the effect of the medium intensity coughing technique during subcutaneous low molecular weight heparin injection on pain severity and individual satisfaction in general surgery patients.

## Method

### Study design and study locus

This is a prospective, quasi-experimental study performed between March and June 2022 in the General Surgery Clinic of a university hospital in the Marmara Region of Turkey.

### Study sample

A convenience sample was used, which included participants, who were consecutively accessible over a period of time and who met the eligibility criteria. The eligibility criteria for the sample were being over the age of 18, having a doctor’s prescription for subcutaneous LMWH 0.6 mL treatment and not yet having begun treatment. Having no disorder that could affect pain perception, having no incision, lipodystrophy, or finding of infection at the injection site, having no communication problem, and voluntarily agreeing to participate in the research were also considered as inclusion criteria. Patients who had diabetes mellitus, peripheral vascular disease, etc. which could affect the perception of pain were excluded from the study sample.

**Figure 1 -:**
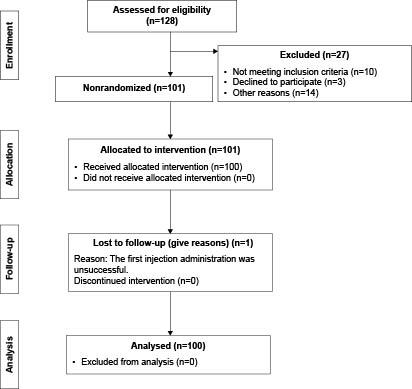
Flowchart of the Transparent Reporting of Evaluations with Nonrandomized Designs (TREND) shows the number of the participants through each stage of the study

The size of the research sample was decided statistically with the program G*Power 3.1.7. In the analysis to determine the sample size, it was calculated that a total of 80 patients was needed for a significance level of 0.05 and 80% power in determining the effect size of pain intensity as 0.9 in the comparison of two types of injection method. Finally, the research sample included 100 patients, 44 female and 56 male, who had been prescribed a subcutaneous LMWH injection once in 24 hours (once a day) by a doctor. The Strengthening the Transparent Reporting of Evaluations with Nonrandomized Designs (TREND) guidelines was also used for this study^(31)^. Figure1 shows the research flow chart according to TREND.

### Data collection instruments

A Patient Description Form, a Visual Analog Scale, and the Visual Individual Satisfaction Scale were used in collecting the research data.

#### Patient Description Form

This form included contained four questions for getting information about the patients’ age, gender, height, weight and body mass index (BMI). BMI was classified according to the World Health Organization (WHO) classification: below 18.50 kg/m^2^ was classified as underweight, 18.5–24.99 kg/m^2^ as normal weight, 25–29.99 kg/m^2^ as overweight, and 30 kg/m^2^ or more as obese^(32)^.

#### Visual Analog Scale (VAS)

In evaluating the severity of pain felt by the patients during subcutaneous injections, a 10 cm long vertical VAS was used, on which one end represented no pain, and the other end the worst possible pain. The VAS is a commonly used pain assessment scale in clinical settings. Its validity and reliability in measuring the severity of pain in adults have been demonstrated^(33)^. Pain severity measurements were evaluated in millimeters.

#### Visual Individual Satisfaction Scale (VISS)

During the administration of the injection, an evaluation was made to determine individuals’ satisfaction using the Visual Individual Satisfaction Scale, which consisted of a vertical 10 cm scale with “I’m very satisfied” written at one end of it and “I’m not at all satisfied” at the other. The Visual Individual Satisfaction Scale incorporated the characteristics of the well-known Visual Analog Scale (VAS). VISS is a valid scale widely used in adults to assess individual satisfaction^(34)^.

### Ethical considerations

The necessary permission to conduct the research was obtained from the Clinical Research Ethics Committee of the Medical Faculty of Bursa Uludag University (Decision No: 2022-6/18). Also, oral and written approval was obtained from the patients for their voluntary participation in the research, after information had been given.

### Data collection

After the voluntary participation of the patients included in the research had been secured, their descriptive characteristics were collected from the Patient Description Form. After that, they were given information on the use of the VAS and VISS. Each patients received two injections. All injections were given once a day and were administered each morning at 11:00. All injections were administered to the upper part of the right or left arm on the outer area by the same researcher. The reason for performing the injections was that the patients were in the surgical clinic, most had incisions in the abdominal area, and the nurses in the clinic preferred the upper arm as the injection area. Figure2 shows the steps of the administration of the standard subcutaneous LMWH applied to all patients.

**Figure 2 -:** Injection administration procedure

Region of application Volume Needle size Syringe type Air lock Wipe Insertion angle Aspiration procedure Injection duration	Outer side of upper arm 0.6 mL 27 gauge Prefilled single dose 0.3 mL air lock inserted Area cleansed with alcohol and allowed to air-dry before needle insertion 90^o^ Not performed 10 seconds

The injections were randomly allocated to injection sides for one of two injection methods. The two different injection techniques applied included in the research are given below.

*Injection method I:* Before receiving the subcutaneous LMWH injection, the patient was asked to take a deep breath and cough once with moderate intensity to empty her/his lungs. After 10 seconds, the patient was asked to take a deep breath and cough a second time with the same intensity^(20),(22)-(23)^. During the second cough, the needle was inserted into the tissue. The patient’s injected arm was prevented from moving during coughing.

*Injection method II:* During the subcutaneous LMWH injection, patients were not asked to perform any action, and the injection was given by the standard technique.

After each injection, another researcher, who had no prior knowledge of which injection technique was used, immediately assessed pain intensity and satisfaction using the VAS and the VISS and recorded them on the data collection form with the numerical value equivalent to the point which the patient had marked. While the study was being conducted, the necessary measures were taken to prevent the individuals from affecting each other, such as not allowing patients taking part in the study to see the method applied to other patients, and not including in the study at the same time patients who were staying in the same room.

### Statistical analysis

Statistical analysis of the research data was performed with the statistics package IBM SPSS 22.0 (IBM, Armonk, NY, USA). Numerical data was examined by the Shapiro-Wilk test as to whether it showed normal distribution. Distributions of descriptive information on patients obtained as a result of the study were given as means and SDs. Since the data was found to be normally distributed, the non-parametric Wilcoxon signed rank test was used in dependent groups, and the Mann-Whitney U test and Kruskal Wallis-H test were used for two independent variables. The level of statistical significance was determined as p < 0.05.

## Results

It was found that 56% of the patients participating in the research were male, their mean age was 60.48±12.76 years, 29% were in the clinic for a cholecystectomy operation, 38% were overweight, and their mean BMI was 26.69±5.56 kg/m^2^ Table1.

**Table 1 -:** Findings concerning patients’ descriptive characteristics (n=100). Bursa, Turkey, 2022

Variables	n	%
**Gender**		
Female	44	44.0
Male	56	56.0
**Body Mass Index (BMI)**		
Underweight	5	5.0
Normal	35	35.0
Overweight	38	38.0
Obese	22	22.0
**Mean age (years)**	60.48±12.76	
Mean BMI* (kg/m^2^)	26.69±5.56	

*Body Mass Index

Table2 shows the mean scores of pain severity and satisfaction of the patients after the injection. According to this, the mean pain severity of patients after an injection administered by the coughing technique was 25.89±20.77 mm, while the mean pain severity perceived after an injection given by the standard method was 52.30±25.51 mm. Also, patients’ mean satisfaction levels after the injection were found to be 92.09±8.02 mm after injection by the coughing technique, and 76.19±15.24 mm after injection by the standard technique. Statistical analysis showed that there was a statistically significant difference between patients’ mean scores on pain severity and satisfaction levels after injections performed by the two methods (p<0.05, Table2).

**Table 2 -:** Comparison of pain severity and satisfaction levels of patients by injection method (n=100). Bursa, Turkey, 2022

	Method I	Method II	Statistical value
VAS* (mm)	25.89 ± 20.77	52.30 ± 25.51	Z^†^ = -8.108, p= 0.000
VISS^‡^ (mm)	92.09 ± 8.02	76.19 ± 15.24	Z^†^ = -8.104, p= 0.000

^*^Visual Analog Scale; ^†^Wilcoxon signed ranks test; ^‡^Visual Individual Satisfaction Scale

Table3 shows the mean scores of pain severity and satisfaction level of the patients after injections administered by the two methods according to gender and body mass index. According to the statistical analysis, the severity of pain of female patients after injections administered by the coughing technique was at a statistically significantly higher level than that of male patients (p<0.05, Table3). However, no difference was found between the mean pain severity of male and female patients injected by the standard technique, or between their reported satisfaction levels after injection by either technique. Also, it was found that the variable of the patients’ body mass index did not affect to a statistically significant extent their mean pain severity or satisfaction level scores after injection by either method (p<0.05, Table3).

**Table 3 -:** Comparison of patients’ pain severity and satisfaction levels according to gender and body mass index (n=100). Bursa, Turkey, 2022

	Pain Severity		Satisfaction Level	
	Method I	Method II	Method I	Method II
**Gender**				
Female	30,45 ± 20,14	52,84 ± 26,51	91,00 ± 9,62	78,15 ± 13,25
Male	22,30 ± 20,73	51,87 ± 24,92	92,94 ± 6,47	74,65 ± 16,58
	Z* = -2,181 p = 0,029	Z* = -0,400 p = 0,689	Z* = -0,704 p = 0,481	Z* = -1,075 p = 0,282
**BMI^†^**				
Underweight	21,20 ± 19,56	65,00 ± 18,01	92,80 ± 5,26	67,20 ± 22,99
Normal	26,45 ± 22,90	49,51 ± 29,51	93,34 ± 6,90	79,82 ± 13,28
Overweight	25,00 ± 3,28	49,50 ± 24,31	91,44 ± 9,67	73,88 ± 17,53
Obese	27,59 ± 19,50	58,68 ± 21,10	91,04 ± 7,20	76,45 ± 10,75
	K-W^‡^ = -1,309 p = 0,876	K-W^‡^ = -1,017 p = 0,395	K-W^‡^ = -2,045 p = 0,690	K-W^‡^ = -1,192 p = 0,353

*Mann-Whitney U Test; ^†^Body Mass Index; ^‡^Kruskal-Wallis Test

## Discussion

Subcutaneous LMWH injections are an important part of drug administration. One of the basic responsibilities of nurses is to protect patients from preventable side effects by the use of correct techniques(9),(13)^. Taking under control the pain which frequently occurs in the injection area in connection with the administration of subcutaneous LMWH injections will make acceptance of the treatment easier by increasing an individual’s comfort and satisfaction.

Therefore, it is important that there is a need for research into an effective, easily applied technique because of uncertainties concerning which technique to use in order to control pain and increase satisfaction when giving subcutaneous LMWH injections, considering factors such as heavy pressure of work, rapid patient turnover, the large number of patients and the lack of time, especially for healthcare professionals working in surgical clinics. In this regard, it was seen in the results of this study that the medium intensity coughing technique used during subcutaneous LMWH injections significantly reduced patients’ pain severity in comparison with injections given by the standard technique. No studies were found in the literature examining the effect of this technique in the administration of subcutaneous LMWH injections, but it was seen that it had been considered in connection with the management of pain developing in connection with other invasive nursing procedures.

In a study on the subject, it was concluded that the coughing technique was effective in reducing pain during peripheral intravenous catheterization, and was an equivalent technique to the method of complex distraction^(22)^. Similarly, a study investigated the effect on pain of techniques such as coughing, squeezing a stress ball, or blowing into a spirometer while performing peripheral intravenous catheterization on healthy adults. It was found in the study that the lowest mean pain severities resulted from the application of the medium intensity coughing technique^(23)^. In studies on the subject conducted with pediatric patients, it is reported that the coughing technique was an effective method in reducing pain during vaccination^(19),(21)^ and during the collection of blood samples^(20)^. The results of these studies are seen to be similar to our research. It is stated in the literature that the coughing technique reduces pain by providing an antinociceptive effect working in parallel with the study mechanisms relating to baroreceptor activation^(20)-(21)^ and vagus nerve stimulation^(23)-(28)^. It has also been reported that this technique is helpful in pain management, as it relies on the most frequently used attention diversion technique in bringing pain under control^(23),(29)^. From this information, it can be said that the coughing technique is effective in reducing the pain arising from subcutaneous LMWH injections, depending on the potential mechanisms mentioned above.

As a result of this study, the conclusion was reached that reported satisfaction levels after subcutaneous LMWH injections performed with the technique of getting patients to cough were significantly higher than after injections administered by the standard technique. It is reported that patient satisfaction is one of the important indicators of the quality of nursing services, and is accepted as a desired result of health services^(35)^. Studies have found that the mechano-analgesia technique, when compared with the standard technique of reducing the pain of subcutaneous LMWH injections, increased patient satisfaction^(6)^, and that the manual pressure method increased comfort levels^(12)^. Along with reduction of pain, an increase in patient satisfaction is expected. Although the methods used to control pain are different, it is seen that the techniques which were effective in reducing pain in the results of the studies mentioned above also increased satisfaction with the injection. Evaluated from this aspect, the increase in satisfaction with the injection with the coughing technique, with which patients reported less pain in our study, was assessed as a likely result.

In a study in which the effect of mechano-analgesia and cold application on bruising, pain and patient satisfaction relating to subcutaneous heparin injection was investigated, it was concluded that the mean pain severity scores of female patients were significantly higher than those of male patients, but that the variable of gender did not affect the level of patient’satisfaction^(6)^. Similarly, it was found in the present study that the mean pain severity of female patients following an injection administered by the coughing technique was significantly higher than that of male patients, but that the variable of gender did not affect the level of satisfaction of patients after an injection. In some studies in the literature in which different techniques were used to reduce the pain felt during subcutaneous heparin injections, a significant correlation was found between pain severity and gender^(9)^, but in others, it was concluded that there was no such correlation^(12),(36)-(37)^. It is thought that these differences between the studies mentioned above and the results of our study may arise from differences in the subcutaneous injection techniques with patients included in the study groups.

It was found as a result of this study that the variable of patients’ body mass index did not significantly affect the mean scores of pain severity and satisfaction level after injection by either method. Examining studies in the literature, it was found that they concluded that the variable of body mass index had no effect on the pain severity^(12),(37)^ and satisfaction level^(6),(12)^ connected with subcutaneous heparin injection. It is seen that the findings of our study are similar to the results of these studies. However, one study investigated the effect of manual pressure applied for different lengths of time following subcutaneous heparin injection on pain and bruising, it was concluded that the mean pain severity of overweight and obese patients was significantly greater than that of underweight or normal weight patients^(9)^. In our study, it was found that the pain severity of obese patients was greater than that of underweight, normal weight and overweight patients, although this did not reach the level of significance. It has been reported in other studies that obese individuals experience pain more intensely^(38)-(39)^. Also, it has been reported that because pain receptors are found in the subcutaneous tissue, stimulation of this region will cause a feeling of pain^(6)-(11)^. Although the results of our study are similar to the literature in this respect, it is though that these differences between studies may arise from differences in the injection technique, the region of the body where the injection was given, and the patients’ mean body mass indexes.

Nurses are often undecided as to what technique to use in order to manage this pain in patients and to increase satisfaction, and there is a need for an effective technique which is easy and simple to use. In this way, this study makes a contribution to the literature on non-pharmacological methods used to reduce the pain of subcutaneous LMWH injections and to increase satisfaction with the injection. An important contribution of the study to the literature and its originality is that it is the first study to assess the use of the coughing technique with subcutaneous LMWH injections.

One of the most widespread complications arising in connection with the administration of subcutaneous LMWH injections is pain. Nurses are often undecided as to what technique to use to manage this pain in patients and to increase satisfaction, and there is a need for an effective technique that is easy and simple to use. In this way, this study contributes to the literature on non-pharmacological methods used to reduce the pain of subcutaneous LMWH injections and increase satisfaction with the injection. Furthermore, considering that it is simple and free to use on patients, this technique can provide great convenience in the clinical field for health professionals, particularly nurses, in terms of pain management via safe subcutaneous heparin injections.

This study has some limitations. The most important limitations are that the results could not be generalized because the subcutaneous heparin injection was applied only to the patient profile in the general surgery clinic, only on the outer side of the upper arm as the application area, and with 0.6 mL as the application dose. For this reason, it is recommended that comparative studies be conducted by repeating the study on different injection sites, with different application doses, and with different patient profiles.

## Conclusion

The results of the current study found that the medium intensity cough technique applied to patients during the administration of a subcutaneous LMWH injection reduced the severity of the patients’ pain and increased the level of satisfaction which they felt with the injection. Also, it was found that the variable of gender affected pain severity relating to the injection, but did not affect the level of individual satisfaction. In addition, it was found that there was no correlation between the variable of BMI and the severity of pain and level of satisfaction.
