# Erratum:Effect of the coughing technique during subcutaneous heparin injection on pain severity and individual satisfaction

**DOI:** 10.1590/1518-8345.0000.3966

**Published:** 2023-06-19

Regarding the article “Effect of the coughing technique during subcutaneous heparin injection on pain severity and individual satisfaction”, with DOI number: 10.1590/1518-8345.6504.3924, published in the Rev. Latino-Am. Enfermagem, 2023;31:e3924, page 1:

Where was written:

Derya Uzelli Yılmaz^ 2^

Fatma Düzgün^ 3^

^2^ Bursa Uludağ University, Health Application and Research Center, Bursa, Turkey.

^3^ İzmir Katip Çelebi University, Faculty of Health Sciences, Department of Nursing, İzmir, Turkey.

Now read:

Derya Uzelli Yılmaz^ 3^

Fatma Düzgün^ 2^

^2^ Bursa Uludağ University, Health Application and Research Center, Bursa, Turkey.

^3^ İzmir Katip Çelebi University, Faculty of Health Sciences, Department of Nursing, İzmir, Turkey.

